# Outdoor education in Canadian post-secondary education: common philosophies, goals, and activities

**DOI:** 10.1007/s42322-022-00102-4

**Published:** 2022-08-24

**Authors:** Morten Asfeldt, Rebecca Purc-Stephenson, Thomas Zimmerman

**Affiliations:** Department of Social Sciences, Augustana Faculty, University of Alberta, AB Camrose, Canada

**Keywords:** Outdoor education, Higher education, Post-secondary, High impact educational practice

## Abstract

Canada has a long history of outdoor education (OE) in sectors including summer camps, K-12, and post-secondary education (PSE). However, previous research has demonstrated that OE is sometimes poorly understood in the PSE sector leading to program closures and limited program development. As a result, scholars have called for national and international collaboration to promote the value of OE including identifying common OE learning outcomes and the theoretical and philosophical underpinnings guiding OE practices. Therefore, the purpose of this study is to identify common philosophies, learning goals, and characteristics of PSE OE in Canada. Results reported here are part of a larger three-phase, exploratory sequential mixed-method project and used a convenience sample of PSE outdoor educators recruited from across Canada. Findings indicate that PSE OE in Canada is influenced by six common philosophies and includes five common learning goals. Overall, this study demonstrates that OE is well aligned with the common goals and missions of PSE in Canada.

## Introduction

According to Passmore (1972), the University of Alberta was among the first universities in Canada to offer for-credit outdoor education (OE) courses with an initial offering in the 1960s. At the time of Passmore’s 1972 nationwide study of OE in Canada, he also identified OE courses at four other universities. However, the first organized OE activity at a Canadian university may have been at McGill University in Montreal, QC by the McGill Outdoors Club. The McGill Outdoors Club is a student club founded in 1936 that continues to offer programs today (McGill Outdoors Club, n.d.). More broadly, OE in Canada has strong roots in the Ontario summer camp movement with the first camps appearing in the early 1900s (Wall, 2009). In the 1960s and 70 s, OE developed further being spurred on by a growing environmental movement resulting in OE appearing in K-12 schools (Raffan, 1996). In the third phase of this larger project, we identified 58 PSE institutions in Canada that offer at least one for-credit course that self-identified as outdoor or adventure education and used an online survey to more deeply understand how the findings from the qualitative phase of this research (Asfeldt et al., 2021) apply in PSE. Recently, Dyment and Potter (2021, p. 16) claimed that OE in PSE is undervalued and poorly understood which often results in OE being “thrown overboard” in the ocean of neoliberalism (Berg et al., 2016). As in other parts of the world, neoliberalism is an increasingly dominant force in the Canadian PSE sector.

In order to counteract this trend of being undervalued and misunderstood, Potter et al., (2012, p. 99) “suggest that outdoor adventure educators must collaborate on national and international levels to promote the value of their discipline.” Similarly, the Outdoor Council of Canada is currently planning a Canadian Outdoor Summit with the goal of “enabling outdoor programs [to] thrive and be appreciated for their true worth” and the “creation of a more unified national outdoor community and begin to remove roadblocks currently affecting outdoor programming” (OCC, n.d.). In addition, Dyment and Potter (2021) identify six lessons learned that they recommend outdoor educators address in order to maintain and strengthen OE in PSE including: having strong evidence to present during program reviews, advocating strategically, and being less humble “in the cut-throat evidence-based world of neoliberal universities” (p. 1). Driven by these concerns, this paper describes common philosophies, learning goals, and characteristics of OE in Canadian PSE. Earlier phases of this project are reported in Purc-Stephenson et al. (2019) and Asfeldt et al. (2021).

## Literature review

### Defining outdoor education

Pinpointing a universal definition of OE is a difficult task. In fact, some scholars (Nicol, 2002; Wattchow & Brown, 2011) claim that no universal definition exists and the quest to develop one is a fruitless endeavor. Inherent in the challenge of defining OE is OE’s interdisciplinary nature that integrates knowledge, skills, and methods from diverse and well-established disciplines such as physical education, biology, psychology, history, and more. This disciplinary diversity is combined with a range of regional and internationally distinct cultural influences such as colonialism and the canoe in Canada (Erickson & Wylie Krotz, 2021), military and character development as seen in the evolution of Scouting and Outward Bound in the UK (James, 1990; MacDonald, 1993), and the Norwegian notion of friluftsliv where nature is seen as the home of culture that grounds OE in many parts of Scandinavia (Henderson & Vikander, 2007). Finally, there is an ongoing debate whether OE is a distinct discipline or a method of teaching (Dyment & Potter, 2015; Quay & Seaman, 2013). Regardless of this ambiguity, in this study, we adopted Henderson and Potter’s (2001) description of OE as a term “denot[ing] the overarching curricular enriching education in the outdoors that include both environmental and adventure education” (p. 69).

We recognize that adopting Henderson and Potter’s definition is a limitation and that some PSE institutions that do not self-identify as outdoor or adventure education have been excluded. Ironically, this limitation may represent a key strength of OE yet contributes to OE being undervalued and misunderstood. That is, one strength of OE is its interdisciplinary and holistic nature which results in OE often being housed in a variety of traditional PSE disciplines and departments such as Physical Education, Outdoor Recreation, Education, and Environmental Studies. These diverse homes suggest that OE may be more frequently viewed as a method than a discipline in PSE in Canada which are typically organized around disciplinary distinctions rather than pedagogical methods. When seen as a method, OE risks being scattered and lost and unable to establish a resilient foundation or endure the changing trends and funding of PSE. This is a difficult conundrum and might suggest that OE take up the challenge of developing a more universal understanding of OE in order to establish a strong foothold in PSE. Regardless of this definitional shortcoming, these findings contribute to a more fulsome understanding of how OE is currently understood by PSE outdoor educators in Canada.

### Outdoor education in Canada

In spite of Canada’s history of OE that spans more than 120 years, it has been nearly 50 years since the last nationwide study of OE (Passmore, 1972). However, there have been many studies that have investigated the outcomes and benefits of specific PSE OE courses and programs in Canada which we will review shortly. But first, we summarize findings from Passmore, highlight ideas from a key conceptual paper (Henderson & Potter, 2001), and provide an overview of earlier stages of this project (Asfeldt et al., 2021; Purc-Stephenson et al., 2019).

Passmore (1972) set out to answer two questions: “How well do our present educational programs meet the needs and interests of all students in our rapidly changing society?” and “What special contribution can well planned, carefully integrated programs of outdoor education and environmental studies make towards the student’s total education?” (p. 7). After months of interviews and observations of K-12 and PSE programs and a variety of community and volunteer organizations and government agencies, Passmore concluded that:

Outdoor environmental education is certainly not the answer to all our educational problems. But there is growing recognition that it is a method of teaching that can add that other important “R” to every subject on the curriculum - *relevance in what we teach about the world in which our young people live.* (p. 61)

Nearly 30 years after Passmore’s study, Henderson and Potter (2001) set out to describe OE in Canada. Overall, they observed that “there is a distinctive Canadian approach to adventure education, not universal, but common across Canadian geography…” (p. 226). They identify six features that characterize OE in Canada: geography, a blended approach, curricular integration, the travel experience, non-professionalization, and being different from the USA.

Henderson and Potter (2001) suggest that geography shapes OE in Canada because outdoor educators live far apart, may not know each other, and rarely gather; programs are uniquely rooted in local geographies and histories; and that wilderness is reasonably accessible. They also offer that OE in Canada employs a blended approach that includes goals and practices of both adventure and environmental education. Similarly, they point to curricular integration as a hallmark of OE in Canada where knowledge, skills, and methods from many disciplines (e.g., history, geography, literature, native studies) are combined into one course or experience. They further claim that the self-propelled remote travel experience is a distinctive pinnacle experience and through this travel experience students develop group and leadership skills as well as connect to Canadian history, identity, and landscape. When Henderson and Potter claim that Canadian outdoor educators are “non-professional”, their point is that OE programs in Canada are commonly driven by one person with a personal and locally informed vision as opposed to a program guided by the curriculum of national or international organizations. Finally, they maintain that OE in Canada is different from many American OE experiences because of the ready access to remote areas, being less focused on certification and accreditation, and a more organic “let the mountains speak for themselves” approach (James, 1980). Henderson and Potter may be correct in their assertions but their work is strictly conceptual. 

In the first phase of this larger project, Purc-Stephenson et al. (2019) conducted a systematic literature review of Canadian OE literature to identify key factors influencing OE in Canada and to describe OE learning outcomes and psychosocial benefits. This review identified eight themes describing learning outcomes and psychosocial benefits: developing outdoor-living skills, risk and challenge, gaining environmental knowledge, personal growth and leadership skills, sense of community, building connections, having fun in nature, and lasting impacts. In addition, Purc-Stephenson et al. created a conceptual model illustrating the process of OE in Canada. This model begins with educator influences (i.e., expertise, relatability, building trust) and includes a multi-faceted curriculum of history, environmental awareness, and outdoor-living skills. This curriculum is explored through experiences that balance exploration and fun with competence and challenge which results in outcomes of personal growth, developing a sense of community, and building connections. These outcomes are facilitated through intentional processing and reflection that produce a variety of lasting impacts.

In the second phase of this project, Asfeldt et al. (2021) report findings from 22 interviews and site visits from OE programs at summer camps (6), K-12 schools (10), PSE institutions (5), and one CEGEP in Canada. CEGEPs (Collège d'enseignement général et professionnel) are one-and two-year programs offered only in the province of Quebec that provide diplomas and certificates. The findings identified five distinct guiding philosophies and values: influential founders (96%), hands-on experiential learning (86%), holistic and integrated learning (68%), journeying through the land (55%), and religion and spirituality (27%). The examination of program learning goals revealed five common goals: building community (91%), personal growth (91%), people and place consciousness (64%), environmental stewardship (55%), and employability and skill development (45%). Investigating the activities of OE programs resulted in the creation of eight activity themes: outdoor-living skills (95%), sport and recreation activities (91%), curricular activities (72%), reflection (59%), environmental education activities (50%), games (50%), arts and crafts (45%), and certification courses (32%). Overall, Asfeldt et al. concluded that “OE in Canada is grounded in experiential learning outdoors that link academic disciplines, and included the added benefit of helping students make connections with the land, its people, and our past” (p. 307).

### Outcomes and benefits

There is a growing body of Canadian research investigating the outcomes and benefits of OE. These outcomes and benefits include lifelong learning, environmental literacy and sustainable education goals, outdoor skill development, personal and social skill development (Asfeldt & Hvenegaard, 2014; Dyment & O’Connell, 2007, Hutson and Weber, 2008, Wigglesworth & Heintzman, 2017), building a sense of community (Asfeldt et. al., 2017; Breunig et al., 2010), outdoor expeditions as holistic integrated learning (Harper & Webster, 2017), and the value of combining outdoor skills training, place stories, and outdoor journeys as a means of enhancing nature place relationships (Mikaels & Asfeldt, 2017). A few Canadian PSE institutions have developed outdoor orientation programs for incoming students and research demonstrates that these programs result in enhanced academic success, personal development, integration into the campus community, and significant life-long learning (Lathrop et al., 2012; Meilleur et al., 2020). One group of researchers have also investigated OE as an alternative to traditional sports hazing (Johnson & Chin, 2015). While not specifically Canadian, there is growing evidence of the personal wellness benefits of spending time in outdoor and nature places (Gray, 2019; Thomsen et al., 2018). These wellness benefits have been further demonstrated in research growing out of the COVID pandemic (Pouso et al., 2021; Soga et al., 2021) and represent a timely and strategic opportunity for OE.

### Challenges and opportunities

In addition to the outcomes and benefits of OE in Canada, a number of scholars, both Canadian and international, have identified challenges and opportunities for OE. For example, Dyment and Potter (2021) investigate why “once-vibrant [OE] programs [have] declined or been closed” (p. 1) and group the challenges faced by PSE OE into three clusters: societal trends and beliefs, high-level leadership and power structures, and personal roles. Their societal trends and beliefs cluster includes the rise in neoliberalism and the scant understanding of OE’s common learning outcomes. The high-level leadership and power structures cluster includes challenges linked to the misalignment of strategic visions with senior leaders' perception of OE, new management change agendas, external reviews that imposed change, reviews that were essentially a fait accompli, and opinions that OE courses are too expensive. The final cluster, personal role, points to the lack of strategic advocacy by OE faculty and staff as a barrier to the success of OE programs. Based on these challenges, Dyment and Potter (2021) recommend that outdoor educators understand the neoliberal agenda, develop strategic alliances with senior leaders, seek leadership roles in university administration, be prepared with evidence to contribute to reviews, strategically advocate for OE programs, and finally, they “question the merit of being overly humble in the cut-throat evidence-based world of neoliberal universities” (p. 1).

Dyment and Potter (2021) also identify opportunities for OE. Specifically, they recommend that outdoor educators emphasize the common learning outcomes of OE that are well aligned with university visions and graduate attributes that employers value and highlight the strong theoretical underpinnings of OE such as place-based education, sustainability, experiential education, environmental education, social justice, and ecoliteracy which are central to many PSE strategic plans. As well, one of Dyment and Potter’s interviewees identifies the wellness benefits of OE as an untapped opportunity that may help OE be more highly valued and understood.

A number of scholars discuss opportunities and challenges related to current social issues. For example, Canadian Lowan-Trudeau (2014) states that experiential environmental education is an opportunity for educators to “foster locally grounded authentic engagement and collaboration between Indigenous and non-Indigenous people and epistemologies…” (p. 363) which can help Canada move towards reconciliation with Indigenous people who live in what we now know as Canada and serve as a means to addressing a number of issues related to privilege and racism more broadly. For example, Meerts-Brandsma et al. (2020)–speaking internationally–points out that while some aspects of OE are “steeped in colonial thought” (p. 2), OE is also an “ideal environment for teaching about privilege” (p. 2).

Finally, many OE scholars (e.g., Beames & Brown, 2016; Roberts, 2016; Wattchow & Brown, 2011) have highlighted the value of common OE practices as effective educational practices. For example, OE includes many of the common features of engaged educational practices leading to student success such as Kuh et al’s (2017) key features of high-impact educational practices (HIEP) (e.g., significant time investment; interactions with faculty and peers; timely feedback; learning through real-world applications; reflective and integrated learning) which are commonly stated priorities and measures of success for many PSE institutions in Canada. For example, common PSE priorities include hands-on learning, service learning, volunteer experience, developing skills such as relationship-building, communication and problem-solving skills, analytical and leadership abilities (Universities Canada, n.d.). These are hallmark characteristics of OE which are supported by a growing body of research. Therefore, as the Canadian PSE moves toward taking learning outside the classroom and into the world, OE has the potential to become a key contributor, and model, in this mission. However, in order for OE to find a place in the future of PSE, OE must be more clearly understood by colleagues, administrators, and government decision-makers. With these challenges and opportunities in mind, we developed three research questions: (1) What are the common philosophies, (2) learning goals, and (3) activities of PSE OE in Canada?

## Materials and methods

### Research design

This study is part of a larger research project that employed a three-phase, exploratory sequential mixed-method approach to examine OE in Canada (Creswell & Creswell, 2018). The first phase involved a systematic review of the literature (Purc-Stephenson et al., 2019) and the second phase was a small, qualitative study to explore the range of issues and to generate ideas (Asfeldt et al., 2021). The present study is the third phase that used a non-experimental descriptive research design. Specifically, we developed a quantitative survey tool based on our previous research and conducted an online survey to a larger sample of OE PSE instructors.

### Sampling strategy

We used purposive sampling (Vehovar et al. 2016) and created a sampling frame to identify PSE institutions in Canada that offered at least one program or a course on OE. We defined PSE as universities, colleges, and CEGEPs. Generally, university programs offer bachelor’s, master’s, and doctoral degrees, whereas colleges offer diplomas, certificates, and trade certifications.

Guided by Henderson and Potter’s (2001) definition of OE, we searched for PSE programs and courses that offered environmental or adventure education in any discipline, and incorporated at least some of its curriculum in the outdoors. We conducted an internet search of PSE institutions using the search terms “outdoor” and “adventure.” We also piloted terms such as “experiential education,” “experiential learning,” “environmental education,” and “field studies” to expand our search; however, these terms proved to be too broad. Therefore, to be included in our sample, a program or course needed to (1) be offered at a PSE, (2) include at least some of its curriculum in the outdoors, (3) include at least one learning goal, and (4) be currently operating or have been operating within the last two years. We excluded campus recreation and other non-credit OE experiences.

### Procedure

For each PSE, we invited one instructor or administrator to complete the survey. In cases where we could identify a specific outdoor program director or course instructor, we sent the invitation directly to them. Otherwise, the invitation was sent to an administrative leader or manager. We sent the email invitations between August and October 2019, which included the study description, consent form, and survey links. The study material was available in both French and English, Canada’s two official languages. We asked that respondents have a minimum of five years of work experience in an OE or related field and be at least 18 years of age. Participants interested in completing the survey clicked a button that stated “I consent to be in this study,” and they were directed to the survey website. The survey took approximately 20 min to complete. Participants had the option to be included in a random draw for a $25 gift certificate to an online outdoor retailer. No reminder emails were sent.

### Materials

The survey was developed based on findings from earlier phases of this project (Asfeldt et al., 2021; Purc-Stephenson et al., 2019) and was piloted by four independent OE colleagues who were not eligible to participate in the study. The survey consisted of 5 sections: participant demographics; program characteristics; program values and philosophies; program learning goals; and program activities.

#### Participant demographics

We collected the demographic information including the participant’s age, gender, education level, years working in OE, years in their current position, and their job title.

#### Program characteristics

We asked 16 questions to understand the nature of the OE programs and courses offered at each institution. The questions included: what province their program/course was located; when the program/course was first established; how the program/course was organized (e.g., undergraduate/graduate, major/minor); year level of courses (i.e., first, second, third, fourth, and/or graduate); and time of year courses were offered (i.e., fall/winter/spring/summer). We also asked whether the courses involved a day program (e.g., regularly spending time outside during normal class time), taking field trips (e.g., class takes a trip), overnight trips (e.g., 1–2 nights away), and/or an expedition (e.g., three or more nights away). For courses involving an expedition, we asked the length of these trips. In addition, we asked how many students generally participated in the program/course each year and if the program/courses required extra fees and the range of those fees. We asked how many instructors generally teach OE courses at their institution. We also asked if their program/course included an Indigenous learning objective, and if so, they were asked to explain what that entailed.

#### Program values and philosophies

Based on our previous research that identified seven values and philosophies commonly underlie OE programs in Canada (Asfeldt et al., 2021), we asked participants to indicate the extent to which these seven values and philosophies influenced their program. The seven values and philosophies included: (1) the vision and values of the program’s founder, (2) a hands-on experiential learning approach (e.g., combines content, experience, and reflection), (3) a holistic integrated learning approach where disciplines (i.e., biology, history, physical education) are blended, (4) religious traditions (e.g., Christianity, Judaism), (5) spiritual connections (e.g., making connections to people and the natural world), (6) self-propelled wilderness travel experience, and (7) educational philosophers (e.g., Dewey, Kolb). Participants responded using a 5-point Likert scale with response options ranging from 1 (strongly disagree) to 5 (strongly agree). Next, we asked participants which two philosophies were *most essential* to their program.

#### Program learning goals

Based on our previous research that found OE programs/courses in Canada have five common learning goals (Asfeldt et al., 2021), we asked participants to indicate the extent to which these five learning goals were part of their program/course. The learning goals included: (1) building community (i.e., promote teamwork and relationships), (2) employability (i.e., students gain practical work experience), (3) environmental stewardship (i.e., students learn sustainable practices), (4) people and place consciousness (i.e., students learn about the location, people, historical significance), and (5) personal growth (i.e., students develop in character, leadership, and self-awareness). Participants responded using a 5-point Likert scale with response options ranging from 1 (strongly disagree) to 5 (strongly agree). Next, we asked participants which two learning goals were *most essential* to their program.

#### Program activities

Our previous research identified 33 common OE program activities (Asfeldt et al., 2021). To understand the breadth and most common activities in PSE OE courses/programs, we asked participants which of the 33 activities were included in their program/course. These activities included outdoor travel activities (e.g., hiking, canoeing, and dog-sledding), outdoor-living skills (e.g., camping, cooking, and building a campfire), nature studies, journal writing, as well as certification courses and safety training. Next, we asked participants to specify which three activities were most common in their program.

### Data analysis

We used SPSS to calculate the descriptive statistics for each survey question. We used Wilcoxon signed-ranked order tests to determine possible differences among program philosophy and program learning goals (Zar, 1999). Open-ended responses were reviewed and thematically analyzed into categories.

## Results

### Participant characteristics

We received 25 responses to our 58 invitations. However, three surveys were excluded due to excessive missing data. Therefore, the final dataset included 22 surveys (37.9% response rate). Of these, the majority of the participants were male (*n* = 16, 72.7%), who were approximately 49 years old (*M* = 49.2, *SD* = 10.2), and had worked in OE for about 22 (*M* = 21.7, *SD* = 11.9). Participant characteristics are presented in Table 1. As not all participants answered every question, the counts may not total 22 and the percentages may not equal 100.

**Table 1:** Participant Characteristics

Variable	*N* (%)
Age (*M, SD*)	49.42 (10.145)
Gender	
Male	16 (72.7)
Female	5 (22.7)
Non-binary	1 (4.5)
Education	
High School diploma	0
College diploma	1 (4.5)
Bachelor’s degree	3 (13.6)
Master’s Degree	8 (36.3)
Doctorate degree	10 (45.5)
Years in OE (*M, SD*)	21.70 (11.859)
Position Job Title	
Director	2 (9.1)
Teacher/Instructor	4 (18.2)
Coordinator	3 (13.6)
Professor	11 (50)
Manager	2 (9.1)

### Program characteristics

Participants reported on 22 separate OE programs. We divided Canada into 6 regions: West (BC), Prairies (AB, SK, MB), Ontario (ON), Quebec (QC), Atlantic (NB, NS, PEI, NL), and North (YT, NT. NU). As Table 2 shows, responses from Ontario were over-represented and responses from Quebec were under-represented.

**Table 2:** Program Locations in the Sample

Region	Number of Survey Invitations Sent (%)	Number of Survey Responses Received (%)
West	14 (24.1)	4 (18.2)
Prairies	9 (15.5)	5 (22.7)
Ontario	11 (19.0)	7 (31.8)
Quebec	16 (27.6)	1 (4.5)
Atlantic	5 (8.6)	2 (9.1)
North	3 (5.2)	2 (9.1)
TOTAL	58 (100)	21 (37.9*)

^*^One respondent did not report region

Table 3 shows that nearly all OE programs were established between 1970 and 2009 (*n* = 16). Most OE courses were offered at either the first-year (*n* = 14, 63.6%) and second-year level (*n* = 15, 68.2%). The type of OE course format varied, with most programs offering more than one format. For example, 20 participants (91%) noted that their program offered outdoor classroom courses whereby students worked outdoors during class time, and 20 participants (91%) noted their program offered courses with an expedition. The length of the expeditions were approximately 9 days long (*M* = 9.1, *SD* = 6.44), and ranged from three days to 26 days.

**Table 3:** Program Characteristics

Variable	N (%)
Year program established	
1970–1979	5 (23.7)
1980–1989	0 (0)
1990–1999	6 (27.3)
2000–2009	5 (23.7)
2010–2020	3 (13.6)
Program type	
Undergrad minor	3 (13.6)
Undergrad major	7 (31.8)
Undergrad dip/certificate	8 (36.4)
Postgraduate dip/certificate	3 (13.6)
Masters	5 (22.7)
Doctorate	3 (13.6)
Year of course	
First year	14 (63.6)
Second year	15 (68.2)
Third year	11 (50)
Fourth year	9 (40.9)
Graduate course	6 (27.3)
Semester/Term	
Fall (Sept-Dec)	21 (95.5)
Winter (Jan-April)	20 (90.9)
Spring/Summer (May–August)	16 (72.7)
Number of OE instructors (*M, SD*)	9 (11.23)
Average number of students (*M, SD*)	87 (92.4)
Type of OE format	
Outdoor classroom (class takes place outside)	20 (90.9)
One Day Field Trip (class day trips)	18 (81.9)
Overnight Trips (outing of 1–2 days/nights)	19 (86.4)
Expedition (outing of 3 + days/nights)	20 (90.9)
Do you students pay extra fees	
Yes	15 (68.2)
No	7 (31.8)
Approximate cost (*M, SD*)	956.7 (1,287.24)
Average expedition length in days (*M, SD*)	9.1 (6.44)
Type of Indigenous learning component included	
Field trip	1 (4.5)
Guest speaker/workshop	1 (4.5)
History/Traditions	4 (18.2)
Integrated	5 (22.7)
Land-based learning	4 (18.2)

### Program philosophy

**Fig. 1:**
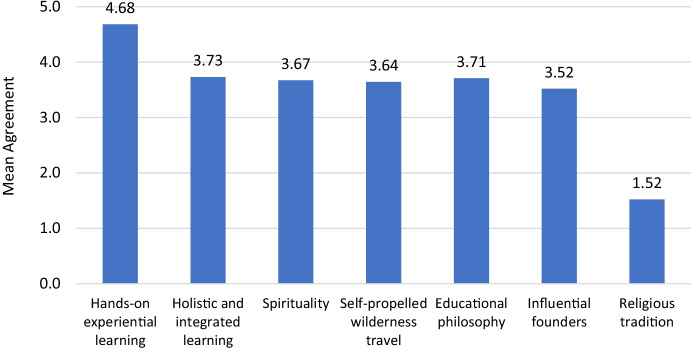
Influential Program Values, Principles, and Philosophies

All OE courses and programs reported that they are influenced by a number of values, principles, and philosophies. As Fig. 1 shows, participants agreed or strongly agreed that hands-on experiential learning was the most influential philosophy guiding their OE program/course (*M* = 4.68, *SD* = 0.89) whereas religious traditions was the least influential (*M* = 1.52, *SD* = 1.08). A series of Wilcoxon signed-rank tests showed that hands-on experiential learning was rated significantly higher than all the other philosophies (all *p*s < 0.05) and that religious traditions was rated significantly lower than all other philosophies (all *p*s < 0.05). When asked to identify the top two most influential philosophies, 20 participants (91%) identified hands-on experiential learning and 8 (36%) identified self-propelled wilderness travel.

### Program learning goals

**Fig. 2:**
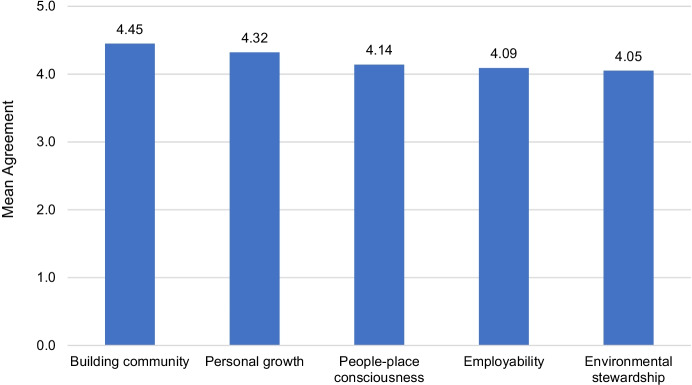
Central Program Learning Goals

Participants reported that their programs/courses have numerous learning goals rather than a single focus. As displayed in Fig. 2, participants agree that all five learning goals were important in their OE program/course. A series of Wilcoxon signed-rank tests revealed no significant differences between the program learning goals (all *p*s > *0.05*). However, when asked which two learning goals were most essential to their program/course, 13 participants (59%) identified personal growth and 9 participants (41%) identified employability learning goals. Environmental stewardship was the least essential learning goal identified (*n* = 3, 13.6%).

Over two-thirds (*n* = 15, 68.2%) of the participants reported that their program/course included an Indigenous learning component; two participants noted there were plans to integrate Indigenous learning components into OE courses but nothing had been implemented yet. We reviewed the open-ended responses and thematically organized them into two broad categories: deeply integrated learning and surface-level learning. About half of the programs (*n* = 7) embedded Indigenous perspectives and decolonization curriculum in their OE courses whereby students had an opportunity to listen to Indigenous guest speakers, interact with elders, participate in a workshop lead by an Indigenous guest speaker (e.g., blanket ceremony), visit a community or school, engage with course readings written by Indigenous scholars. The other half of the programs (*n* = 8) tended to include a single Indigenous learning component to an existing OE course. In these courses, the learning focused on providing an introduction to cultural and historical awareness, and the learning components included having a guest speaker, participating in a workshop (e.g., students make leather winter footwear), learning about traditional shelter styles, travel methods, and trade.

### Program activities

Based on a list of 33 activities, participants reported that their program/course incorporated an average of 14 activities (*M* = 14.2, *SD* = 5.6), ranging from three activities to 22 activities. The most frequently included activities in PSE OE program/courses were camping (90.9%), making campfires, canoeing, and hiking (all at 86.3%), nature studies, orienteering, and safety training (all at 72.7%), cooking, and snowshoeing (both at 68.1%), and journal writing (31.8%). No participant identified archery, caving, or horseback riding. Table 4 presents the activities, which we categorized into seven thematic groups for ease of communication.

**Table 4:** Outdoor Education Program Activities

Activity Category	Description	% of programs/courses incorporating the activities
Outdoor-living Skills	Activities aligned with camping such as safety, camp set-up, cooking, fire building and in some cases hunting and fishing	95.5%
Self-Propelled Travel Methods	Physical activities that taught students technical skills of traveling outdoors such as biking, canoeing, climbing, dog-sledding, hiking, and snowshoeing	95.5%
Work Experience and Certification Courses	Courses including volunteering, and activities leading to certification such as canoeing, and safety training	81.8%
Environmental Education Activities	Immersing learners in the natural environment such as nature walks, pond studies, and birding	81.8%
Games	Group games intended to be fun, interactive, and collaborative	50.0%
Reflection	Processing activities that encouraged students think critically using practices such as journaling, group debriefing, and discussion	77.3%
Creative products	Activities where students participated in making hand-made products (e.g., sewing, beading)	18.2%

## Discussion

This research provides a number of insights regarding the common philosophies, learning goals, and activities of PSE OE in Canada that may be helpful in promoting the value of PSE OE. For instance, there has been a recent emphasis in the Canadian PSE setting on providing increased opportunities for experiential interdisciplinary learning that is guided by critical reflection on theoretical and philosophically sound educational practices (For the Public Good, 2016; Gillis et al., 2017; Regehr, 2013). This trend is partly in response to universities and colleges feeling the need to “re-emphasize the importance of teaching and learning” (Tight, 2018, p. 62) to satisfy criticism that they are not serving undergraduate students well or preparing them adequately for an increasingly interdisciplinary workforce that is tasked with solving societal challenges that require inter-and trans-disciplinary solutions (Gillis, et al., 2017). In spite of these concerns and criticisms, Gillis et al., found that instruction at Canadian universities remains largely discipline-specific and that undergraduate students lack opportunities for program-based transdisciplinary experiential learning. This research points to OE in Canada being commonly guided by trans and interdisciplinary experiential learning. For example, hands-on experiential and holistic integrated learning that includes more than content knowledge but also spiritual aspects of learning that are rooted in experiences of place are important philosophies the drive OE in Canada. For the OE community, this is likely intuitive knowledge that has been known for decades and affirms that Henderson and Potter’s (2001) claim that OE in Canada commonly includes a blended approach (adventure and environmental education), curricular integration (knowledge, skills, and methods from many disciplines), and self-propelled remote travel experiences (experiential place-based learning) is correct. Further, these findings support Passmore’s (1972) conclusion that outdoor education is a form of teaching that helps bridge the gap between theory and practice and the classroom and the world.

Dyment and Potter (2021) indicate that OE often struggles “when the strategic aims of senior leadership teams did not align with those of OE” (p. 7). However, these findings demonstrate considerable alignment between common pedagogical values and goals of PSE in Canada and OE such as hands-on experiential, holistic integrated, and place-based learning. These findings may be helpful as outdoor educators follow Dyment and Potter’s recommendation to engage in more evidence-based strategic advocacy demonstrating the alignment between OE and PSE strategic plans.

Dyment and Potter (2021) report that “many high-level university administrators lacked understanding of, and thus appreciation for, OE’s robust achievable learning outcomes” (p. 7) yet in this study, respondents’ rating of OE learning goals point to OE being well-aligned with the goals of PSE, governments, and employers. For example, the learning goals of building community and personal growth are particularly important to emphasize in light of a recent Conference Board of Canada (Giammarco et al., 2021) report claiming that “despite strong employer demand for social and emotional skills” (p. 2), these skills are not being prioritized by Canadian PSE. Given the growing research that consistently identifies social and emotional skills such as building community, communication and leadership skills, teamwork, resilience, and personal growth as achievable learning outcomes of OE, OE appears well suited to make a meaningful contribution to PSE strategic goals and missions. In addition, the COVID pandemic has increased awareness of the benefits of spending time in outdoor and nature spaces on mental health and wellbeing and warrant strategic emphasis by outdoor educators in PSE.

The central role of employability as a learning goal is notable. Outdoor education students often gain certifications and work experience that prepare them for OE-related employment as well as many other transferable social and emotional skills. These employment training experiences and outcomes are well-aligned with common PSE agendas and are perhaps an aspect of OE that warrants greater emphasis (Dyment & Potter, 2021). One approach to doing this would be tallying the number of days students are in the field, engaged in leadership and internship opportunities, and the certifications gained as well as clearly describing how students learn essential skills such as problem-solving, building community, and communication that research demonstrates are common outcomes of OE. 

Issues related to environmental sustainability and people and place consciousness are pressing issues facing Canadians. Specifically, Canadians are increasingly impacted by climate change and face a variety of unique and local environmental challenges. At the same time, Canadians have a commitment to reconciliation with Indigenous Canadians (Truth and Reconciliation Commission [TRC], 2015). OE has a long history of enhancing environmental awareness and sustainability through engaging students in environmental hands-on experiences both locally and during remote travel experiences (Asfeldt & Hvenegaard, 2014; Passmore, 1972; Van Matre, 1974; Wattchow & Brown, 2011). As these results demonstrate, remote travel is common in PSE OE in Canada and provides students with hands-on integrated experiences that are well suited to enhancing people and place consciousness. If well designed and facilitated, these characteristics of OE can help students understand Canada’s troubled and colonial past that has led to many harms, both social and environmental. In this way, OE is well equipped to take up the challenges of our current time to contribute towards moving Canada towards a more socially just and sustainable future.

The activities included in PSE OE programs in Canada vary considerably. There are many factors that likely influence this variety including the location of the program (e.g., rural vs urban; access to oceans or rivers or mountains). Regardless, it is notable that the most commonly included activities are camping and either canoeing, hiking, skiing, or kayaking. The combination of camping and an activity that facilitates remote self-propelled travel experiences makes sense because it prepares students for experiential hands-on experiences of holistic integrated learning in remote spaces which are a common foundational value of OE in Canada. In addition, these activities combined with the self-propelled travel experience are central to the experiential achievement of building community, personal growth, people and place consciousness, employability, and environmental stewardship which are common goals of OE in Canada. Therefore, while viewing OE activities in isolation may lead senior leaders and colleagues to perceive OE as lacking pedagogic rigor because of the association of these activities with recreational and leisure pursuits, it is important to understand that the activities are a purposeful means to an end. That is, the activities are a means of practicing and honoring the philosophical and theoretical foundations that ground OE such as experiential and place-based learning, and for achieving the central learning goals of OE. Therefore, in order to fully understand OE, it is critical to understand the integration and critical connections between OE’s common philosophies, learning goals, and activities.

### Implications

This study builds upon a growing body of research that has a number of potential implications for OE in the Canadian PSE sector. First, as Potter et al., (2012) and Dyment and Potter (2021) point out, pressing financial constraints and increasing neoliberal trends are putting pressure on many aspects of PSE that have resulted in the decline and closure of some OE programs. To combat this trend, Potter et al (2012) claim that if OE is to establish a stronghold in PSE, OE “must collaborate on both national and international levels to promote the value of their discipline” (p. 99) and OE would be wise to “develop a coherent message as to the value and benefit of their programmes, research and curriculum” (p. 116). In addition, Dyment and Potter (2021) indicate there is scant understanding of OE’s achievable learning outcomes, that there is a perception that OE is poorly aligned with strategic visions of PSE, and that faculty need to advocate more strategically for OE and be able to produce strong evidence regarding the value and benefits of OE. While this research does not claim that the philosophies, learning goals, and activities identified here are *universal* aspects of *all* PSE OE programs in Canada, this research is a step towards developing a coherent message about *common* pedagogical values, learning goals, and activities in many PSE OE programs in Canada and can be used as evidence to support OE in PSE. This is especially true when this research is coupled with previous research regarding the practices, outcomes, and benefits of OE in Canada and internationally.

Second, as Meerts-Brandsma et al. (2020) point out, OE has partly evolved from a colonial past. We urge outdoor educators to think critically about their intentional and unintentional practices and messages to ensure that OE does not perpetuate colonial and racist stereotypes and myths, particularly during remote travel experiences. In fact, this study demonstrates that OE is well-suited to make an effective contribution towards decolonization and reconciliation and addressing privilege more generally because of its hands-on, holistic, interdisciplinary, group focused, and place-based traditions.

Third, Potter, et al. (2012) and Dyment and Potter (2021) question the merit of being overly humble regarding the values and benefits of OE. The findings of this research serve as a contribution to the growing evidence that demonstrates that OE commonly provides the type of engaged, innovative, active, and experiential group and place-based learning that facilitates a wide range of learning objectives that colleges and universities identify as priorities.

### Limitations

We recognize that our sampling relied on PSE self-identifying programs and courses using the term “outdoor” or “adventure” in the program title, course title, or course description. As such, it is possible that we did not identify all programs and courses that offered OE. While a gap always exists between the target population and the sampling frame applied (Till & Matei, 2016), we attempted to limit this gap by expanding our search terms and by sending our list of identified OE programs and courses to several Canadian PSE OE colleagues for validation. We also cross-checked our list against data from another study examining outdoor leadership training programs in Canadian PSE (Williams-Orser, 2021). Williams-Orser used inclusion criteria narrowly focused on outdoor leadership, and identified 33 PSE institutions in Canada. While our samples overlap, we identified more institutions (*N* = 58) as our inclusion criteria was broader.

Another limitation is that our study does not include equal representation from all regions of Canada. However, these findings combined with the findings of the earlier phases of this larger project (Asfeldt et al., 2021; Purc-Stephenson et al., 2019) do provide substantial evidence that there are some common–though not universal–philosophies, learning goals, and activities that are shared by many PSE OE programs in Canada. In addition, given the challenges of defining OE, we have likely excluded programs and courses that share similar philosophies, goals, and activities with those included in this study yet do not self-identify as outdoor and/or adventure education.

### Future research

The findings of this project point to a long list of interesting and useful questions. For example, examining the alignment of common OE educational philosophies and learning goals with those of individual college and university missions, values, and strategic plans may help academic leaders recognize the contribution that OE programs are making towards institutional and student success. Similarly, identifying best practices and opportunities (local, national, and international) for OE as a means for addressing social and environmental issues, reconciliation, and personal well-being may inspire academic leaders to see a specific role for OE in PSE. Further, more specific research to understand the specific Indigenous learning goals of OE in Canada and the methods and activities employed to achieve those learning goals would be timely. Also, investigating and profiling OE as an example of a HIEP (Kuh et al., 2017) may encourage academic colleagues to see OE as a long-standing model for active, innovative, and engaged teaching and learning rather than a discipline and practice that lacks academic rigor or a strong theoretical and pedagogical foundation.

## Conclusion

In this aspect of our larger project investigating OE in Canada, we narrowed our focus to OE in Canadian PSE and asked three questions: (1) What are the common philosophies, (2) learning goals, and (3) activities of PSE OE in Canada? Our findings suggest that PSE OE in Canada is guided by a small number of philosophies, most programs have a variety of learning goals with the most common goals pertaining to community building and personal growth, and frequently included activities related to outdoor living (e.g., camping, fire-building, hunting) and self-propelled travel methods (e.g., hiking, canoeing). Dyment and Potter (2021) and Potter et al. (2012) call for a collaborative effort among OE educators to promote the value of OE. Our findings contribute to that call and suggest that PSE OE in Canada provides a range of unique learning opportunities for students to address disciplinary topics and emerging issues (e.g., social and emotional skill development, Indigenous perspectives, climate change) in an immersive setting that is well-aligned with commonly stated PSE goals and visions.

## Data Availability

Not applicable.
